# Application of the EVEX resource to event extraction and network construction: Shared Task entry and result analysis

**DOI:** 10.1186/1471-2105-16-S16-S3

**Published:** 2015-10-30

**Authors:** Kai Hakala, Sofie Van Landeghem, Tapio Salakoski, Yves Van de Peer, Filip Ginter

**Affiliations:** 1 Dept. of Information Technology, University of Turku, Turku, Finland; 2 Turku Centre for Computer Science (TUCS), Turku, Finland; 3 Dept. of Plant Systems Biology, VIB, Ghent, Belgium; 4 Dept. of Plant Biotechnology and Bioinformatics, Ghent University, Ghent, Belgium; 5 Genomics Research Institute (GRI), University of Pretoria, Pretoria, South Africa; 6 The University of Turku Graduate School (UTUGS), University of Turku, Turku, Finland

**Keywords:** Text mining, Event extraction, Network construction, Large-scale data, Distributed vector representations of words

## Abstract

**Background:**

Modern methods for mining biomolecular interactions from literature typically make predictions based solely on the immediate textual context, in effect a single sentence. No prior work has been published on extending this context to the information automatically gathered from the whole biomedical literature. Thus, our motivation for this study is to explore whether mutually supporting evidence, aggregated across several documents can be utilized to improve the performance of the state-of-the-art event extraction systems.

In this paper, we describe our participation in the latest BioNLP Shared Task using the large-scale text mining resource EVEX. We participated in the Genia Event Extraction (GE) and Gene Regulation Network (GRN) tasks with two separate systems. In the GE task, we implemented a re-ranking approach to improve the precision of an existing event extraction system, incorporating features from the EVEX resource. In the GRN task, our system relied solely on the EVEX resource and utilized a rule-based conversion algorithm between the EVEX and GRN formats.

**Results:**

In the GE task, our re-ranking approach led to a modest performance increase and resulted in the first rank of the official Shared Task results with 50.97% F-score. Additionally, in this paper we explore and evaluate the usage of distributed vector representations for this challenge.

In the GRN task, we ranked fifth in the official results with a strict/relaxed SER score of 0.92/0.81 respectively. To try and improve upon these results, we have implemented a novel machine learning based conversion system and benchmarked its performance against the original rule-based system.

**Conclusions:**

For the GRN task, we were able to produce a gene regulatory network from the EVEX data, warranting the use of such generic large-scale text mining data in network biology settings. A detailed performance and error analysis provides more insight into the relatively low recall rates.

In the GE task we demonstrate that both the re-ranking approach and the word vectors can provide slight performance improvement. A manual evaluation of the re-ranking results pinpoints some of the challenges faced in applying large-scale text mining knowledge to event extraction.

## Introduction

Our participation in the BioNLP Shared Task (ST) of 2013 was mainly motivated by the question whether large-scale text mining resources could provide supporting information to existing event extraction systems. To this end, we have consulted our previously implemented text mining resource, EVEX, which covers all publicly available literature from PubMed and PubMed Central (PMC OA) [1]. We participated in two subchallenges of the ST'13, implementing different strategies on top of EVEX for each task. For the GE task, additional features for event extraction were generated by mining the EVEX documents in addition to those available through the gold-standard GE datasets. By contrast, our submission to the GRN task relied solely on the information in EVEX, bypassing the need to retrain a new text mining system specifically for this task.

Most earlier event extraction systems have utilized information from a single sentence while extracting potential events [2-5]. Several studies have subsequently expanded this textual context to surrounding sentences through coreference resolution technigues [6,7]. However, to our knowledge, our entry to the ST'13 [8] is the first study on exploiting large-scale information extraction of known interactions to infer more reliable and consistent predictions on new articles. In this paper, we describe our research on different ways of aggregating mutually supporting information across different documents in an attempt to increase the event extraction performance.

In the following sections, we first introduce EVEX as the underlying text mining resource, and then summarize the methods we developed specifically for the GRN and GE task entries [8]. Further, we describe additional experiments conducted after the official ST evaluation, such as the usage of distributed vector representations for the GE challenge and the implementation of a novel machine learning (ML) based conversion system for the GRN task. Finally, we discuss the performance of our methods both for the official ST entries as well as for the novel experiments, providing a detailed error analysis to offer more insight into the challenge of incorporating large-scale text mining results to a specific event extraction task.

## EVEX

EVEX (http://www.evexdb.org) is a text mining resource which focuses on biomedical event extraction and gene interactions, covering the whole biomedical literature available in PubMed and PubMed Central Open-Access archives [1]. The gene and protein mentions included in EVEX are identified with the BANNER named entity detector [9] and the events and interactions connecting these mentions are extracted with the TEES event extraction system [10]. TEES is a natural language processing system which extracts complex, nested biomolecular events from research articles using state-of-the-art supervised learning techniques. Specifically, EVEX relies on the version of TEES released after the Shared Task 2011 [10], trained with the ST'11 GE data.

To enable effortless summarization of the event extraction data across various articles, EVEX provides event generalizations, where equivalent gene and protein mentions are detected [11]. For instance, the canonicalization algorithm deals with small lexical variations by removing non-alphanumerical characters (e.g. 'Esr-1' to 'esr1'). In the *canonical *generalization the events with the same event type and equivalent canonicalized arguments are subsequently grouped together. Similarly, the *Entrez Gene *generalization utilizes the GenNorm system [12] to assign taxonomic classification and Entrez Gene identifiers to gene mentions and groups the events based on this knowledge. A more coarse-grained *family-based *generalization is achieved through homologous families by aggregating candidate interologs and regulogs. The prediction quality of each generalized event is represented by a confidence score automatically derived from the TEES classification scores.

Whereas the event generalizations aggregate events based on the similarity of the gene and protein mentions, the EVEX network interpretation provides an additional level of abstraction by converting complex event structures into pairwise gene/protein interactions and representing these relations as a typed, directed network. Such a network enables much easier analysis and integration with external resources than complex event structures [13]. In previous work, the EVEX network data was shown to contribute significantly to integrative network analyses, and precision rates of 58.5% were obtained both for human data as well as for plant-specific research [14].

## GRN task

### Motivation

The EVEX resource allows to automatically infer regulatory biomolecular networks from literature. To evaluate the performance of this inference mechanism, we have participated in the Gene Regulatory Network (GRN) challenge of the Shared Task 2013. This challenge is designed to address interactions related to the well-known process of sporulation in *Bacillus subtilis*.

As will be detailed in the following sections, we have used the EVEX data exactly as it is publicly available. It is important to note that this results in a more challenging setting and likely affects performance negatively. However, we believe the reported performance will provide a better insight into the applicability and generalizability of text mining systems in general.

### EVEX data conversion

We have first extracted the network formalism of all canonical events in EVEX (see *EVEX *section). Within the large-scale resource, there are over 4 million articles containing such relations. Restricting to *Bacillus subtilis *would result in 17 thousand articles with relevant data. By contrast, the original GRN dataset covers only 172 articles (training, development and test sets combined). Interestingly, each relation extracted from EVEX has an associated confidence value which represents the likelihood of an event being correctly extracted by the text mining methodology underlying the resource.

In a second, non-trivial step, we need to match the gene symbols in EVEX to those in the GRN data. As the symbols in EVEX are computationally generated, they are not fully compatible to the manually curated GRN data. To bridge this gap, we have extracted all tagged gene symbols in the gold standard GRN data, and converted them to a canonicalized version similar to those present in EVEX. This set can then be matched directly to the EVEX data, removing relations between canonical gene symbols that were not encountered in the GRN data. Note that this procedure still allows us to retain relevant interactions found in articles outside the GRN dataset.

The GRN data further provides a standardization of the gene symbols, linking for instance 'sigmaB' to its unique *gene identifier *'sigB'. The above procedure allows us to link also the EVEX canonical symbols to their corresponding GRN identifiers.

Lastly, a conversion between the original EVEX event type and the desired GRN relation type is required. For the official ST'13 GRN entry, we have designed a rule-based mapping which interprets the semantics of the EVEX events and transforms them to GRN types (Table 1). The final set of converted predictions are then filtered to ensure a coherent network structure. In the following sections, we will detail the filtering step first, and further introduce a newly implemented ML-based alternative which covers both the conversion as well as the filtering process.

**Table 1:** Original rule-based conversion of EVEX event types to the GRN interaction types.

EVEX type	GRN type
Binding	Binding
Regulation* of Transcription	Transcription
Regulation* of Gene expression	Transcription
Positive regulation of Any*	Activation
Negative regulation of Any*	Inhibition
Regulation of Any*	Regulation

The table is traversed from top to bottom, and the first rule that matches is applied. Regulation* refers to any type of regulatory event, and Any* refers to any other non-regulatory event type. Because the EVEX Binding type, unlike the corresponding GRN Binding, is symmetrical, the EVEX data is split into two candidates per instance. Because some GRN types do not have an equivalent type in the EVEX resource, such as 'Requirement' and 'Promoter', these types could not be addressed by our work.

### Relation filtering

As a first filtering step, the compatibility between the predicted relation or event type (e.g. Binding) and the entity types (e.g. Gene or Protein) is determined. From the GRN training data and the challenge guidelines, we have constructed a set of rules which capture the expected values of each relation type (Table 2). For instance, a Binding is supposed to happen between a protein (Target) and a gene (Agent), thus successfully excluding protein-protein interactions. The application of these rules to the EVEX-converted predictions ensures compatibility with the GRN challenge. At times, these rules are even more restrictive than the original guidelines, in which case they were found to have a beneficial effect on precision.

**Table 2:** Original rule-based entity-type filtering of event predictions.

GRN event type	Possible target types	Possible agent types
Interaction.Binding	Protein	Gene

Interaction.Transcription	Protein, PolymeraseComplex	Gene, Operon

Interaction.Regulation Interaction.Activation Interaction.Inhibition	Protein, PolymeraseComplex	Gene, Operon, Protein, ProteinComplex

Only those events for which both arguments (target and agent) have a correct entity type, are retained in the result set.

To further adhere to the GRN guidelines, only one edge should be predicted between a specific *Agent *and *Target*. This was implemented by only retaining the edge with the most specific type, thus preferring Transcription over Regulation for instance. Additionally, while this is allowed by the guidelines, we decided to select only the event with the highest confidence value when there would be a contradiction (e.g. Inhibition and Activation). Further performance assessments on the GRN training data guided us to retain 'Mechanism' edges (Transcription and Binding) only when a regulatory edge could not be identified.

Finally, the events corresponding to the EVEX Binding type, were found to align more often to the GRN Transcription type in the training data. Consequently, they were thus systematically refactored as such, but only after the filtering by entity type was performed.

### ML-based alternative

As described above, our original GRN entry applied two main rule-based systems to obtain the final predictions; one concerned with the EVEX-to-GRN event type mapping, and one for the entity-type filtering of event predictions. In experiments conducted after receiving the official ST results, we have implemented a novel ML-based system that attempts to replace both rule-based systems by learning the mapping and filtering directly from the training data.

To this end, we have used the C-SVC libSVM classifier [15] with an RBF kernel. For the type conversion task, 5 classes of features were generated from EVEX for each Agent-Target pair with at least one predicted event between them:

• Event count per event type and in total.

• Event count for the reverse Target-Agent pair.

• Maximal event confidence per event type and for all.

• Maximal event confidence for the reverse Target-Agent direction.

• Whether or not the 'reverse' maximal confidence is higher than the forward direction.

A separate SVM was trained for each GRN interaction type individually, and the optimal parameters of the RBF kernels (*C *and *γ*) were selected by a grid search using 5-fold CV on the training data.

This classification step was then combined to the rule-based entity-type filtering as described in *Relation filtering *section, resulting in a hybrid system. Finally, we also attempted to replace those filtering rules by adding more feature types to the feature vector:

• The type of the Agent entity (e.g. gene, protein, ...).

• The type of the Target entity.

### Performance results

The performance of our original rule-based method, as applied on the GRN training data, is depicted in Table 3. As could be expected, the best recall rates (42%) are achieved when running the analysis without filtering on entity types and on all EVEX articles. As this set is not limited to *Bacillus subtilis *research, it will also contain homologous relations from closely related species. The corresponding relaxed F-score (41%) is relatively high, but the relaxed and strict Slot Error Rate (SER) scores are not acceptable (1.23 and 1.56 respectively), as they should be below 1 for decent predictions.

**Table 3:** Performance measurement of different system settings on the GRN training data.

Method	Dataset	SER	F	Rel. P	Rel. R	Rel. F	Rel. SER
TC	All EVEX data	1.56	8.86	39.29%	**41.98**%	40.59%	1.23
TC, ETF	All EVEX data	1.15	11.53	59.74%	35.11%	**44.23**%	0.89
TC, ETF	*B. subtilis *PMIDs	0.954	**20.81**	71.43%	22.90%	34.68%	**0.86**
TC, ETF	GRN PMIDs	**0.939**	17.39	**80.00**%	18.32%	29.81%	**0.86**

The SER score is the main evaluation criterion of the GRN challenge. The relaxed precision, recall, F and SER scores are produced by scoring the predictions regardless of the specific event types. TC refers to rule-based EVEX-GRN type conversion, ETF to rule-based entity type filtering. The ML-based methods are not shown because they are trained on this dataset.

The implementation of the entity type filtering results in an increase in relaxed precision from 39% to 60%, while at the same time the relaxed F-score obtains a maximum score of 44% and the (strict) SER score improves to 1.15. Further improvements of SER score are achieved by limiting the data to *Bacillus subtilis *articles (0.954). When limiting further to only articles within the GRN dataset, the best SER score of 0.939 is obtained, as well as the best performing measure of relaxed precision rate (80%).

The newly implemented ML-based methods can not be correctly evaluated on the training portion of the GRN dataset, as this data is used for feature engineering, parameter selection and training the classifier. For this reason, we have evaluated both the original rule-based system as well as the novel ML-based methods on the development set of the GRN challenge (Table 4). In terms of SER score, a small performance improvement can be obtained on the development set when using the Hybrid system compared to the original rule-based system. However, when also substituting the rule-based entity filtering ('ML-full'), performance drops. We conclude that the machine learning module is not able to learn the entity-type filtering rules accurately from the training data.

**Table 4:** Performance measurement of different system settings on the GRN development set.

Method	Dataset	SER	F	Rel. P	Rel. R	Rel. F	Rel. SER
TC, ETF	GRN PMIDs	1.00	11.90%	70.59%	**17.91%**	**28.57%**	0.896
Hybrid	GRN PMIDs	**0.985**	5.19%	**90.00%**	13.43%	23.38%	**0.881**
ML-full	GRN PMIDs	1.12	2.27%	57.14%	**17.91%**	27.27%	0.955

For the original rule-based system, only the best setting is included here (cf. Table 3). 'Hybrid' refers to the hybrid ML system with rule-based ETF, and ML-full refers to the classifier with the full feature set.

Table 5 summarizes the results of our two best systems applied on the test set and compares them to the results of the 4 other participants in the GRN challenge. For the official submission, we applied the rule-based run that achieved the lowest SER score (0.939) on the training portion. It scored last place out of 5 participants with a SER score of 0.92 and a relaxed score of 0.81. It is important to note that our rule-based system was thus not overfitted on the training data, as the performance on the test data in fact outperforms the best result on the training data.

**Table 5:** Official GRN performance rates of all participants (first 5 rows), as well as the test result of the Hybrid ML-based system.

	SER	Relaxed SER
University of Ljubljana	0.73	0.64
K.U.Leuven	0.83	0.66
TEES-2.1	0.86	0.76
IRISA-TexMex	0.91	0.60
EVEX (orig)	0.92	0.81
EVEX (hybrid)	0.94	n/a

The evaluation service made available to perform additional test results did not present the relaxed SER scores.

In a parallel effort by colleagues, GRN event annotations were generated by a retrained version of the TEES classifier [16], which resulted in a SER score of 0.86. Remarkably, this score is only 0.06 points better than our original rule-based system which required no retraining, was based upon a version of TEES trained on the GE formalism, and used computationally predicted gene symbols instead of the provided gold-standard entities. Additionally, the algorithms used to generate the data in EVEX were optimised on F-score instead of SER score, and our results clearly show that these metrics are not interchangeable (Table 3 and Table 4). In conclusion, we believe that even though we were not able to obtain top-performing results, the relatively small difference in performance to the retrained TEES submission illustrates that large-scale text mining resources generalize relative well, and that they do not necessarily need to be retrained to be applicable for gene regulatory network construction.

Considering the ML-based methods which were designed after the official evaluation, the hybrid system could obtain the best results on the development set (Table 4). Unfortunately, this did not translate in a gain on the final test dataset (SER score of 0.94).

### Error analysis

#### Missing predictions

Even though the official results on the test set do not specify precision and recall rates, the results on the training and development test strongly suggest that our method has a particularly low recall rate. This could not be easily remedied during method development by adjusting the parameters in favour of recall, as that would severely penalize precision and ultimately the SER score.

**Figure 1:**
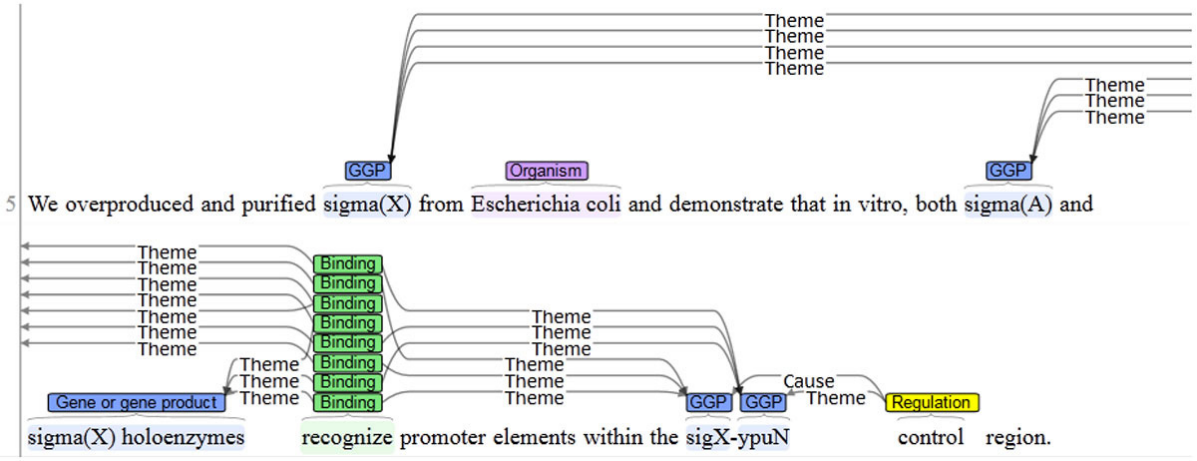
**Incompatible entity span annotation**. Example of entity annotations in EVEX, which are incompatible with the GRN challenge. In this case, the sigX-ypuN operon was not annotated as such by BANNER, but instead two separate gene/gene products (GGPs) were defined.

In an effort to better understand our relatively low recall rates, we have manually analysed the false negative (fn) predictions of our original rule-based system applied to the training dataset. Of the 117 fn predictions, 12% could be attributed to a missing BANNER entity, i.e. a gene/protein name that was simply not tagged as such in the entity recognition step of the EVEX pipeline. A further 11% was caused by incompatible BANNER entities, which could not be directly mapped onto the GRN entities. For instance, the GRN data for PMID:9139908 defines **sigX-ypuN **as an Operon entity, while the BANNER tool annotates **sigX **and **ypuN **as separate gene/protein entities (Figure 1). As a result, two Transcription events with this operon as Target can not be found with our methodology that uses the original EVEX predictions.

**Figure 2:**
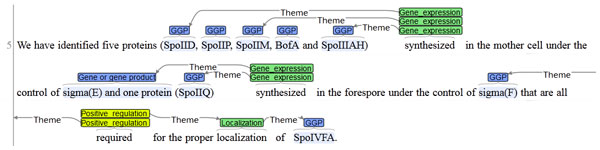
**Missing event structure**. Example of a missing event structure, resulting in a false negative instance for the GRN challenge. In this case, the long sentence and the intermittent subsentences have prevented the event extractor to recognise the requirement relation with all its arguments.

Further, 59% of the missing predictions could be attributed to a false negative TEES prediction, i.e. an event that was not annotated during the event prediction step, even though the entities were correctly tagged. For instance, the GRN data for PMID:15752199 specifies 5 Requirement interactions between spoIID, spoIIP, spoIIM, bofA, and spoIIIAH as Agent on the one hand, and spoIVFA as Target on the other hand. While all 6 entities were annotated as gene/protein, the Requirement relation could not be deduced by TEES, probably because of the relatively long sentence and intermittent subsentences (Figure 2). Further, the wrong interpretation of the structure of this particular sentence led to 8 more missing GRN interactions, showing the drastic influence on performance of one wrongly parsed sentence.

**Figure 3:**
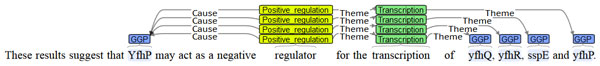
**Wrongly predicted event type**. Example of a wrongly prediction GRN interaction type. In this case, the crucial word 'negative' is not taken into consideration, resulting in a positive regulation of transcription rather than a negative one, which would have led to the correct GRN type 'inhibition'.

Additionally, 15% of the GRN interactions lacking from our predictions were due to the prediction of a wrong event type. For instance, in PMID:10463184 the predicted event correctly linked the tagged entities together, but the crucial word 'negative' was missed in the description of the interaction ('negative regulator for the transcription'). As a result, the event was thus interpreted as a positive regulation in EVEX or, in GRN terms, as an Activation interaction (Figure 3). In a strict evaluation setting, this sentence would thus result in 4 fn results (missing Inhibition relations) as well as 4 fp results (incorrect Activation relations).

Finally, 3% of the fn interactions were due to an incorrect mapping of the gene symbol to the standardized GRN format. For instance, the tagged entity 'forespore-specific sigma factor sigma(F)' in PMID:10767540 could not be linked to the GRN Gene identifier 'sigF'.

#### Wrong predictions

The precision rates obtained by our methodology were encouraging; the application of our original rule-based system to the training dataset only resulted in 16 false positive (fp) predictions. 25% of these fp predictions could be attributed to an incorrectly predicted event structure, for instance by wrongly interpreting which entity was the Agent argument and which the Target. A further 62.5% of the incorrect predictions were due to a wrongly predicted event type, either by the original event extraction module, or by the conversion from EVEX to GRN. We have tried to resolve this issue by implementing the ML-based algorithm for type conversion as described in *ML-based alternative *section, but with limited success. Finally, one false positive was deemed correct after manual verification, but it originated from a sentence outside the GRN data, and in one case a correctly predicted negation context was not taken into account during the conversion.

In conclusion, we believe that the conversion of EVEX output to the GRN formalism will be difficult to further improve upon. General performance could probably only be enhanced by further improving the individual modules for entity recognition and event prediction in the EVEX pipeline.

## GE task

Our GE submission builds on top of the TEES 2.1 system as available just prior to the ST'13 test period [16]. Our approach is based on a post-processing step where the predictions from the unmodified TEES are re-ranked and filtered in an attempt to remove false events, thus improving the quality of the final output. In the official evaluation results published by the ST organizers, this step is shown to lead to a minor increase of 0.23 pp in F-score compared to unprocessed TEES output (Table 6). This resulted in the *first rank *out of 10 participants, with TEES ranking second.

**Table 6:** Official test set results of the five best performing GE participants, in percentages.

	P	R	F
EVEX	58.03	45.44	50.97
TEES-2.1	56.32	46.17	50.74
BioSEM	62.83	42.47	50.68
NCBI	61.72	40.53	48.93
DlutNLP	57.00	40.81	47.56

All in all, 10 teams participated in this task.

Our main motivation for this entry was to assess how bibliome-wide event extraction data, in particular, the EVEX resource can be used as a source of valuable domain knowledge in identifying the most reliable event predictions.

### Event re-ranking

We re-rank the output of TEES using SVM*^rank^*, a formulation of Support Vector Machines adopted to optimize ranking instead of classification [17]. In contrast to a basic linear SVM classifier, SVM*^rank ^*allows the user to define meaningful subsets of instances (query sets). In this experiment we have specified a query set to include all events within a given sentence and thus the SVM is not trying to learn ranking across sentences.

During the re-ranking a numerical score is assigned to each event candidate and subsequently events below a certain threshold score are removed. However, this score should be examined only in the given query set, i.e. within a single sentence, and thus we do not use a pre-defined, data-wide threshold value. Instead, we regard the problem of deciding the threshold as a regression task and set the threshold for each sentence independently. To accomplish this, we train a linear SVM regressor with the SVM*^light ^*package [18].

The re-ranker treats each event as an independent example and the features describe the event itself, its local context, i.e. other events in the same sentence, and most importantly the bibliome-wide knowledge about similar event representations. The features derived from the event capture information about the prediction confidence of the underlying TEES system, as well as structural information such as the paths from the root trigger to all argument leaves. Similar information is aggregated from the other events seen in the same sentence.

The bibliome-wide information is gathered from the EVEX resource by searching similar events from other documents. As EVEX provides multiple levels of abstraction the given event can be compared to the other known events in varying grade of similarity. These features include information on occurrences of the exactly same event description found in literature, events with partially overlapping descriptions, e.g. events with the same event type and at least one equivalent argument gene or protein, and through the EVEX network abstraction also events sharing the same pairwise interpretations. For all of these cases, the counts and confidences of the matching occurrences are encapsulated in the generated features. In this experiment we ended up using the *canonical *and *Entrez Gene *generalizations (*EVEX *section), i.e. the argument gene and protein mentions are considered equivalent if they have only minor lexical differences or they are mapped to the same Entrez Gene identifiers, respectively.

Unlike the re-ranker, the threshold regressor receives features describing individual sentences and the set of events extracted from them. The features used in the re-ranking and threshold regression are described in further detail in Hakala et al. [8].

### Training phase

A set of event descriptions with their known ranking is required for the training of the re-ranker. As our goal is to use these rankings for classification, true positive events are given rank 1 and false positive events rank -1. Since the ST training data includes only true positive event annotations, we generate our own false positive event structures for the training. This can be done naively by generating events with randomly selected trigger words and arguments from the sentences. This approach, however, would lead to unrealistic event representations easily distinguishable from the true positive events. Instead, we apply TEES to the training data and evaluate these predictions against the gold standard, which allows us to gather a set of false positive events TEES is not able to differentiate from the true positives. The re-ranker is subsequently trained on these event representations.

Similarly the re-ranker is again applied to the TEES predictions on the training data to obtain realistic ranking scores for threshold regression training. The optimal threshold for each sentence is then defined as the re-ranker score of the last event to be included in the final output in order to achieve the maximal F-score within the given sentence.

Some sentences may include exclusively either false positive or true positive events in which case no threshold can be selected with the aforementioned approach. For sentences containing only false positive events, the threshold is defined as the highest seen re-ranking score in this sentence increased by value of 0.2, empirically established by optimizing the performance on development set. Likewise, for sentences with only true positive events the lowest score decreased by 0.2 is used.

### Error analysis

Our approach consistently outperforms the TEES system on both the development and the test sets, although with only a modest overall improvement: in the official evaluation our system achieves 1.71 pp gain in precision over TEES for a 0.73 pp loss in recall. As TEES is already biased towards higher precision, this leads only to a small improvement of 0.23 pp in F-score.

Examining the results per event type, as summarized in Table 7, reveals that the performance improvement gained from the re-ranker originates from the regulation events, with a 2.38 pp precision increase for a 0.67 pp recall drop, resulting in an increase of 0.36 pp in F-score for this category. In contrast, for simple events, protein modifications and binding interactions the re-ranking approach in fact decreases the performance. However, as the regulatory events are by far the largest group of events, the performance gain in this category surpasses the decrease in others, resulting in an overall improvement.

**Table 7:** Performance of our system for different event types compared against the TEES system, in percentage points, according to the official GE test results.

	#	P	R	F
Simple events	833	-0.08	-0.36	-0.23
Protein mod.	191	+0.09	-2.09	-1.12
Binding	333	+0.43	-1.20	-0.44
Regulation	1944	+2.38	-0.67	+0.36

All	3301	+1.71	-0.73	+0.23

To obtain deeper insight of the influence of the re-ranker and threshold regressor on the overall results independently, we analyse these modules in isolation.

To assess the maximal attainable performance of the re-ranker without the influence of the threshold regressor, we define an oracle threshold for each sentence. This threshold is adjusted to obtain the maximal F-score within each sentence and can thus be used to evaluate the performance of the re-ranker. However, for sentences with only false positive predictions, the oracle simply obtains the decisions from the gold standard, rendering the ranking irrelevant. This, in turn, leads to very optimistic performance estimate, mainly due to sentences with a single false positive event prediction, which constitute 15.9% of all sentences with at least one predicted event. For a more realistic estimate we therefore establish, in addition to the aforementioned *best case *oracle threshold, a *worst case *oracle, not allowed to filter events from sentences including only false positive predictions. To obtain the correctness of each event we use our own performance measure implementation and evaluate the performance on the development set.

Table 8 summarizes the performance evaluation with the oracle thresholds and suggests that even with the worst case oracle, our re-ranker has the potential to improve the performance substantially: precision is increased by 9.5 pp for a 0.8 pp recall drop, resulting in an increase of 3.3 pp in F-score compared to the baseline TEES system. With our current threshold regressor, however, only a 1.7 pp increase in precision for a 0.8 pp drop in recall is obtained, leaving most of the potential gain unrealized.

**Table 8:** Performance of our current system in the GE task in contrast to TEES, the best case (B-C) and worst case (W-C) oracles with re-ranked output, as well as the worst case oracle with randomized rankings (averaged over ten runs).

All events	P	R	F
B-C oracle (re-ranked)	81.32	39.61	53.27
W-C oracle (re-ranked)	54.92	39.61	46.02
W-C oracle (random)	51.06	39.19	44.34
Current system	47.15	39.61	43.05
TEES	45.46	40.39	42.77

**Single-arg. events**			

B-C oracle (re-ranked)	81.37	50.58	62.38
W-C oracle (re-ranked)	56.09	50.58	53.19
W-C oracle (random)	52.73	50.00	51.33
Current system	48.66	50.44	49.53
TEES	47.16	51.09	49.04

**Multiple-arg. events**			

B-C oracle (re-ranked)	81.02	16.83	27.87
W-C oracle (re-ranked)	48.61	16.83	25.00
W-C oracle (random)	42.66	16.75	24.05
Current system	39.64	17.12	23.91
TEES	37.57	18.17	24.50

The best case oracle surpasses its worst case counterpart by 26.4 pp in precision suggesting that significant performance improvements can be gained from the sentences with solely incorrect predictions. Indeed, sentences with exclusively one or two false event predictions constitute 26% of all sentences with predicted events, influencing substantially the overall performance.

As the oracle is able to obtain the optimal threshold directly from the gold standard annotations, it can lead to a performance gain even with a randomized ranking. As an example, sentences with only one false positive and one true positive prediction are to be ranked correctly by chance in half of the cases leading to the oracle being able to exclude the false positive event by setting the threshold between these two predictions. Thus, to evaluate the real influence of the re-ranker, the re-ranked worst case oracle results are compared against random ranking. Comparing the randomized ranking with the original TEES output sheds light on the influence of an oracle to define the threshold values, whereas the difference of the re-ranked and randomized output reveals the true influence of the re-ranker. The difference between the ranked and randomized results is more evident for multiple-argument events (5.95 pp of precision) than for events with a single argument (3.36 pp of precision). We speculate this to be at least partially caused by the richer feature representation of the multiple argument events, which also benefits from the EVEX network interpretation.

In Table 9 we summarize our overall development set performance using the official evaluation service. We study the performance on single-argument events(column *1-arg*) and multiple-argument events (column *N-arg*) separately, by artificially increasing the re-ranking scores of the contrary event class to always exceed the threshold, thus preserving all TEES predictions in this category. These results confirm that our end-to-end system is able to increase the performance of TEES only on single-argument events. However, the influence of the multiple-argument events on the overall score is negligible due to their low frequency.

**Table 9:** Performance of the system on the GE development set when applied to single-argument events only (*1-arg*), to multiple-argument events only (*N-arg*), and to all events (*Full*).

	TEES	1-arg	N-arg	Full
Simple events	64.43	+0.07	*±*0.00	+0.07
Protein mod.	40.47	+0.06	*±*0.00	+0.06
Binding	82.03	*±*0.00	*±*0.00	*±*0.00
Regulation	30.34	+0.70	-0.14	+0.53

All events	45.04	+0.66	*±*0.00	+0.64

To conclude, the re-ranker appears to be more effective on multiple-argument events, as shown in Table 8, possibly due to the greater benefits from the EVEX based features. However, the results in Table 9 suggest that the end-to-end system performs better on single-argument events. This would imply that the separate parts of the system are optimizing their performance towards different event classes.

### Manual evaluation

Since the ST'13, we have carried out a manual evaluation of 50 false positive predictions, to assess why the gain obtained from the large-scale features did not reach our expectations. This evaluation focused only on events with two or more arguments as they should have benefited the most from the EVEX data. In addition to inspecting the predicted event and its corresponding sentence in the GE dataset, we also evaluated similar interactions from the EVEX resource, including events with lexical variants of the gene or gene product (GGP) mentions, found in articles outside the GE data. We found that 20 out of the 50 false positive predictions had at least one correct mention with exactly the same event structure in EVEX, whereas 5 cases had only false supporting mentions and 25 had no mentions at all. In addition, 12 predicted events were found to have correct mentions which did not match exactly, but still had a similar pairwise interpretation. This means that 32 out of the 50 fp predictions (64%) were found to be biologically meaningful and supported by textual evidence in EVEX, even though that specific relation was not actually mentioned in the GE dataset and thus evaluated as 'wrong'.

As the re-ranker relies not only on the existence of supporting mentions, but also on their counts and confidences, we additionally inspected the number of EVEX mentions supporting the 50 fp predictions, and how many of them were indeed found to be correct. In 5 cases, which all had at least one correct exact match, the number of supporting mentions was over 100 and they were excluded from further evaluation due to practical reasons. From the 15 remaining predictions with correct exact matches, 2 cases were found with more than half of their mentions being false, in addition to the 5 cases where all supporting mentions were false, thus suggesting that a notable amount of noise is present in the mention count features the re-ranker has to utilize. Moreover, 7 cases had the supporting mention with highest confidence wrongly predicted in EVEX, although previous studies have shown the correlation between correctness and the confidence values used in EVEX [19]. These findings may explain why, even though being an intuitive idea, the application of bibliome-wide data back into a single event mention as supporting evidence does not provide large improvements in the prediction performance.

### Other directions explored

As our method proposed in this paper is based on a post-processing stage aiming at removing false event predictions, it is not able to improve the recall of the underlying system, but to only increase the precision. Since most of the state-of-the-art systems, such as TEES, are already biased towards high precision with considerably lower recall, the outcome of such post-processing approach is hardly able to influence the F-score even if the precision is increased substantially. During the shared task we tried to overcome this issue by adjusting the TEES event trigger prediction thresholds in order to over-generate event candidates. This, however, did not result in any performance gains as a large portion of the over-generated events are clearly incorrect. Since the ST'13, we have explored more controlled ways to over-generate events so as to gain the ability to improve the overall recall of the system. In particular, we have assessed whether better results can be achieved by fine-tuning the trigger over-generation for each event type separately. These experiments did not result in adequate improvements, considering the added complexity in the system. Another direction we have explored was to over-generate argument edges, aiming to amend partially correct events by adding missing argument relations. However, this approach drastically decreased the overall performance, for instance an increase of 3.49 pp in recall was paired with a decrease of 10.67 pp in precision.

#### Vector space word representations

To try and enhance our original ST'13 event extraction performance, we have since explored the usage of continuous distributed vector representations of words, namely the state-of-the-art word2vec approach [20]. Word2vec trains a model resembling common neural network language models (NNLMs), yet being computationally efficient and allowing the usage of training data with billions of words. Even though word2vec learns word representations purely from the surrounding context and is thus not aware of syntax, morphology or semantics, the representations have been shown to capture syntactic and semantic characteristics of the words, and they have been successfully applied to various NLP tasks [21]. The usage of word similarities and word clusters in event extraction has been studied previously [22] and just prior to this study the utilization of neural network models in event trigger detection has also been suggested [23]. However, the previously suggested approach is based on older NNLM architectures and the size of the generated vocabulary is thus limited by the computational inefficiency of these models. Furthermore, our goal was to experiment with semantic word representations in all stages of the event extraction pipeline.

TEES relies on a vast amount of features generated from the tokens, stems, part-of-speech tags and dependency parses among other information. As most tokens and syntactic structures occur very rarely in the training corpus, the created feature space is extremely sparse. Our goal in using the vector representations was thus to densify the feature space by describing the words on a more general level, yet retaining their semantics.

Our word2vec model was trained with all PubMed and PubMed Central documents found from the EVEX resource, constituting around 120M sentences. The difference to a full Pubmed and PMC document set is that EVEX only includes those documents with at least one GGP mention detected by BANNER. As word2vec uses an input format where each text span separated with spaces is considered an independent token, GGP mentions such as *'BMP-6 type I receptors' *would be divided into multiple tokens, each having their own vector representation instead of a single representation of the whole GGP. To prevent this from happening, we replaced spaces with underscores in every GGP mention detected by BANNER, thus forming connected spans and enabling word2vec to learn the representation of the whole mention. This pre-processing step wouldn't be possible without the bibliome-wide entity detection provided in EVEX. The vector dimensionality was set to 300 and we used the skip-gram approach. For other word2vec parameters, such as the context window size, default values were used.

In this study, we explored the usage of vector representations in two different ways. In the simplest form, the word vectors themselves were supplied as additional features to TEES. As the second approach, we generated word classes by clustering the word vectors. Clusters were formed for three different word sets: 1,000,000 most common tokens in our vector model, all distinct event triggers in EVEX and a random sample of 1,000,000 gene or protein mentions in EVEX. The clustering was done with the Scikit-learn tool [24], using the Mini-batch k-means algorithm [25], and all sets were divided into 100 and 1,000 clusters.

TEES utilizes a pipeline of three different classification steps. In the first step, TEES detects plausible event triggers, thus resembling a named entity recognition task. In the second step, the argument relations between triggers and GGP mentions are predicted. Since the relations are predicted independently of each other, it is possible that some of the predicted event structures are invalid, e.g. one regulation trigger may have several predicted cause arguments. The third processing step, called unmerging, divides the arguments into valid combinations and tries to filter out wrong events. By default, TEES generates features for a given trigger or argument from the token, stem and part-of-speech tags etc. Similar features are generated for nearby words in the linear context as well as in the dependency graph. The newly introduced vector representations were added in a similar fashion for each word in the linear and dependency graph context, word classes being categorical features and each dimension in the vector space being an independent feature.

The impact of different features on different processing steps can be seen in Table 10. Even though the vector representations show a clear improvement of +0.50 pp in F-score over TEES on the development set when applied to edge detection and event unmerging, their impact on test set performance is detrimental (-0.41 pp). However, for the trigger detection step these features show a modest improvement on both development and test sets, +0.31 pp and +0.16 pp in F-score respectively. The clustering features do not seem to improve performance on this task, showing a decrease of -0.24 pp and -0.92 pp in F-score on the test set, when applied to either trigger detection or edge detection and unmerging.

**Table 10:** Performance comparison of the vector representations of words on the GE development and test set.

	Development			Test		
	**P**	**R**	**F**	**P**	**R**	**F**

TEES	52.49	45.07	48.50	56.32	46.17	50.74
Vectors #1	52.96	45.26	48.81	56.91	46.05	50.90
Vectors #2	53.16	45.45	49.00	55.97	45.71	50.33
Clusters #1	53.09	44.85	48.62	56.41	45.71	50.50
Clusters #2	52.53	45.29	48.64	54.73	45.71	49.82

Vectors #1 uses vector representations in trigger detection, Vectors #2 in edge detection and unmerging step. Clusters #1 uses word clusters in trigger detection and Clusters #2 in edge detection and unmerging step.

### Future work

One problem complicating the threshold regression is the vast amount of sentences including only false positive predictions, as no exact threshold can be set for these sentences during the training phase. We have tried to overcome this issue by training a separate classifier to identify sentences which should not contain any events. This classifier was based on similar features as the threshold regressor, with additional features including bag of words and bag of POS tags. Such sentence filtering was beneficial in excluding obviously incorrect event predictions, when used together with event over-generation, but the gain was not enough to result in overall improvement as the over-generation drastically decreases the precision of the system. As these sentences have a considerable impact on the performance of the system, they should be studied more closely as a future work.

An additional observation made in the manual evaluation was that 9 out of the 50 false positive events evaluated were self-interactions, i.e. *'gene A regulates gene A' *or *'gene A binds to gene A'*. 3 of these cases had supporting evidence in EVEX, but in 2 cases all supporting mentions were incorrect, indicating that TEES is prone to making these kind of errors in the ST'13 data as well as in bibliome-wide data. This suggests that future efforts in improving TEES should be focused on increasing the precision of self-interaction events in particular.

EVEX also offers new opportunities for studying the vector space models. As EVEX includes bibliome-wide GGP mention normalization, it is possible to replace the text spans with the corresponding gene or gene family identifiers. This will allow us to train vector representations that learn the semantic characteristics of the complete gene or gene family profiles, including synonyms and lexical variants. One future direction will be to investigate how a model trained this way will differ from the common fully text-based models and whether this technique can be used to reduce noise in the representations of rarely mentioned GGPs.

Finally, our word clustering could be done with a hierarchical approach for easier assessment of optimal size of word classes.

## Conclusions

In this paper we have summarised our entry to the BioNLP Shared Task 2013 based on the large-scale text mining resource EVEX, and described the further improvements made since.

In the GRN task we have shown that a gene regulatory network can be constructed from the EVEX data without the need to retrain any task-specific text mining methods. The described system performed only 0.06 SER points worse than the corresponding TEES submission specifically trained for the task, warranting the application of generalized text mining resources such as EVEX in network biology settings. In addition to these original findings, in this extended work we have described a novel machine learning approach to replace the rule-based algorithms previously used in our system. This method did not lead to significant performance improvements, but may allow easier adaptation to new task definitions in the future, thus enhancing the applicability of the EVEX resource. We will focus our future efforts on enriching the EVEX resource with more fine-grained event type information beyond the GE task event type definitions. We hope this will enable us to prune some of the false-positive events as well as distinguish more specific event types such as promoter binding (Protein-Gene Binding) and protein-protein interactions (Protein-Protein Binding).

In the GE task, we utilized the bibliome-wide knowledge from EVEX in a re-ranking approach in order to increase the prediction quality of the underlying TEES system. With this experiment we achieved a modest performance gain over TEES, resulting in the first place on the GE task. However, we have demonstrated that the re-ranking approach can increase the overall performance substantially if the per-sentence threshold regressor can be improved. In the manual evaluation conducted since the ST'13, we have pinpointed some of the plausible causes for the lower-than-expected performance, mainly arising from the noisy nature of the text mining data. In particular, a vast amount of the false predictions were verified to have supporting, biologically relevant, statements in the literature, although they were not correct in the given context.

Following the ST'13 we have also experimented with state-of-the-art vector space word representations and shown that they can provide a small performance increase. Although the performance gain was modest when evaluated on the official ST data, we believe that the distributed word representations can be useful in bibliome-wide event extraction, where the variability of the textual content is much higher than in the narrow subset of documents selected for the ST data. The study has identified several future research directions for both approaches.

## Competing interests

The authors declare that they have no competing interests.

## Authors' contributions

KH designed and implemented the GE experiments under the supervision of FG. SVL designed and implemented the GRN experiments, and performed the manual error analysis. FG provided the word2vec models used in the GE task. KH, FG and SVL wrote the manuscript, with feedback from YVdP and TS. All authors read and approved the final manuscript.

## Declarations

The publication costs of this article were funded by the Finnish Academy of Science and the University of Turku.

This article has been published as part of *BMC Bioinformatics *Volume 16 Supplement 16, 2015: BioNLP Shared Task 2013: Part 2. The full contents of the supplement are available online at http://www.biomedcentral.com/bmcbioinformatics/supplements/16/S16.

## References

[B1] Van Landeghem S Björne J Wei CH Hakala K Pyysalo S Ananiadou S Kao HY Lu Z Salakoski T Van de Peer Y Ginter F Large-scale event extraction from literature with multi-level gene normalization PLoS ONE 2013 8 4 55814 10.1371/journal.pone.0055814 PMC3629104 23613707

[B2] Kim JD Nguyen N Wang Y Tsujii J Takagi T Yonezawa A The Genia event and protein coreference tasks of the BioNLP Shared Task 2011 BMC Bioinformatics 2012 13 Suppl 11 1 22759455 10.1186/1471-2105-13-S11-S1 PMC3384256

[B3] McClosky D Riedel S Surdeanu M McCallum A Manning C Combining joint models for biomedical event extraction BMC Bioinformatics 2012 13 Suppl 11 9 22759463 10.1186/1471-2105-13-S11-S9 PMC3395172

[B4] Björne J Ginter F Salakoski T University of Turku in the BioNLP'11 Shared Task BMC Bioinformatics 2012 13 Suppl 11 4 22759458 10.1186/1471-2105-13-S11-S4 PMC3384251

[B5] Vlachos A Craven M Biomedical event extraction from abstracts and full papers using search-based structured prediction BMC Bioinformatics 2012 13 Suppl 11 5 22759459 10.1186/1471-2105-13-S11-S5 PMC3384253

[B6] Yoshikawa K Riedel S Hirao T Asahara M Matsumoto Y Coreference based event-argument relation extraction on biomedical text Journal of Biomedical Semantics 2011 2 Suppl 5 6 22166257 10.1186/2041-1480-2-S5-S6 PMC3239306

[B7] Miwa M Thompson P Ananiadou S Boosting automatic event extraction from the literature using domain adaptation and coreference resolution Bioinformatics 2012 28 13 1759 1765 22539668 10.1093/bioinformatics/bts237 PMC3381963

[B8] Hakala K Van Landeghem S Salakoski T Van de Peer Y Ginter F EVEX in ST'13: Application of a large-scale text mining resource to event extraction and network construction Proceedings of the BioNLP Shared Task 2013 Workshop 2013 26 34

[B9] Leaman R Gonzalez G BANNER: an executable survey of advances in biomedical named entity recognition. Pacific Symposium on Biocomputing Pacific Symposium on Biocomputing 2008 652 663 18229723

[B10] Björne J Ginter F Salakoski T Generalizing biomedical event extraction BMC Bioinformatics 2012 13 suppl. 8 422226192

[B11] Van Landeghem S Ginter F Van de Peer Y Salakoski T EVEX: a PubMed-scale resource for homology-based generalization of text mining predictions Proceedings of the BioNLP 2011 Workshop 2011 28 37

[B12] Wei CH Kao HY Cross-species gene normalization by species inference BMC Bioinformatics 2011 12 Suppl 8 5 22151999 10.1186/1471-2105-12-S8-S5 PMC3269940

[B13] Kaewphan S Kreula S Van Landeghem S Van de Peer Y Jones P Ginter F Integrating large-scale text mining and co-expression networks: Targeting NADP(H) metabolism in E. coli with event extraction Proceedings of the Third Workshop on Building and Evaluating Resources for Biomedical Text Mining (BioTxtM 2012) 2012

[B14] Van Landeghem S De Bodt S Drebert ZJ Van de Peer Y The potential of text mining in data integration and network biology for plant research: A case study on Arabidopsis The Plant Cell 2013 25 3 794 807 23532071 10.1105/tpc.112.108753 PMC3634689

[B15] Chang CC Lin CJ LIBSVM: A library for Support Vector Machines ACM Trans Intell Syst Technol 2011 2 3 27 12727

[B16] Björne J Salakoski T TEES 2.1: Automated annotation scheme learning in the BioNLP 2013 Shared Task Proceedings of BioNLP Shared Task 2013 Workshop 2013

[B17] Joachims T Training linear SVMs in linear time Proceedings of the ACM Conference on Knowledge Discovery and Data Mining (KDD) 2006

[B18] Joachims T Making large-scale SVM learning practical Advances in Kernel Methods - Support Vector Learning 1999

[B19] Van Landeghem S Hakala K Rönnqvist S Salakoski T Van de Peer Y Ginter F Exploring biomolecular literature with EVEX: Connecting genes through events, homology and indirect associations Advances in Bioinformatics 2012 2012 582765 1 12 10.1155/2012/582765 PMC3375141 22719757

[B20] Mikolov T Chen K Corrado G Dean J Efficient estimation of word representations in vector space ICLR Workshop 2013

[B21] Mikolov T Yih Wt Zweig G Linguistic regularities in continuous space word representations Proceedings of NAACL HLT 2013 746 751

[B22] McClosky D Surdeanu M Manning C Event extraction as dependency parsing for bionlp 2011 Proceedings of BioNLP Shared Task 2011 Workshop 2011 Association for Computational Linguistics, Portland, Oregon, USA 41 45 http://www.aclweb.org/anthology/W11-1806

[B23] Zhou D Zhong D He Y Event trigger identification for biomedical events extraction using domain knowledge Bioinformatics 2014 30 11 1587 1594 24489368 10.1093/bioinformatics/btu061

[B24] Pedregosa F Varoquaux G Gramfort A Michel V Thirion B Grisel O Blondel M Prettenhofer P Weiss R Dubourg V Vanderplas J Passos A Cournapeau D Brucher M Perrot M Édouard Duchesnay Scikit-learn: Machine learning in Python Journal of Machine Learning Research 2011 12 2825 2830

[B25] Sculley D Web-scale k-means clustering Proceedings of the 19th International Conference on World Wide Web 2010 177 1178

